# Exploring attitudes and perceptions of patients and staff towards an after-hours co-pay clinic supplementing free HIV services in Kampala, Uganda

**DOI:** 10.1186/s12913-017-2524-5

**Published:** 2017-08-22

**Authors:** Adelline Twimukye, Rachel King, Walter Schlech, Faridah Mayanja Zawedde, Tom Kakaire, Rosalind Parkes-Ratanshi

**Affiliations:** 1 Infectious Diseases Institute, Makerere University, Kampala, Uganda; 2 University of California, San francisco, CA 94105 USA; 3 Dalhousie University, Truro, Canada; 4 Cambridge Institute of Public Health, Forvie Site, Cambridge, CB2 0SR UK

**Keywords:** HIV services, Fee for service, Attitudes, Perceptions

## Abstract

**Background:**

There has been a rapid scale up of HIV services and access to anti-retroviral therapy in Africa over the last 10 years as a result of multilateral donor funding mechanisms. However, in order to continue to expand and to sustain these services it is important that “in country” options are explored. This study sought to explore attitudes and perceptions of people living with HIV (PLHIV) and health care staff towards using a fee-based “after hours” clinic (AHC) at the Infectious Diseases Institute (IDI) in Kampala, Uganda.

**Methods:**

A cross-sectional study design, using qualitative methods for data collection was used. A purposeful sample of 188 adults including PLHIV accessing care at IDI and IDI staff were selected. We conducted 14 focus group discussions and 55 in-depth interviews. Thematic content analysis was conducted and Nvivo Software Version 10 was used to manage data.

**Results:**

Findings suggested that some respondents were willing to pay for consultation, brand-name drugs, laboratory tests and other services. Many were willing to recommend the AHC to friends and/or relatives. However, there were concerns expressed of a risk that the co-pay model may lead to reduction in quality or provision of the free service. Respondents agreed that, as a sign of social responsibility, fees for service could help underprivileged patients.

**Conclusion:**

The IDI AHC clinic is perceived as beneficial to PLHIV because it provides access to HIV services at convenient times. Many PLHIV are willing to pay for this enhanced service. Innovations in HIV care delivery such as quality private-public partnerships may help to improve overall coverage and sustain quality HIV services in Uganda in the long term.

**Electronic supplementary material:**

The online version of this article (doi:10.1186/s12913-017-2524-5) contains supplementary material, which is available to authorized users.

## Background

The number of poeple living with HIV in Uganda (UNAIDS 2015) is estimated at 1.5 million [1, 2]. The most recent robust survey of HIV in Uganda (AIDS Indicator Survey 2011), found that HIV prevalence is highest in the 35–39 age group (12% women, 11% men), with women having higher rates than men at all ages under 44 years [2]. Rates in adolescents aged 15–19 years are 3.7% rising sharply to 5.4% in 20–24 year olds. Despite significant sucess in scaling up HIV treatment services, HIV prevalence remains around 7% [2] and the number of new infections per year remains around 100,000 [3]. Whilst the number of people accessing ART has increased to around 57% of people living with HIV (PLHIV) in Uganda in 2015 [3], World Health Organisation (WHO) recommendations encourage all people with HIV to be started on treatment at the time of diagnosis therefore increasing [4].

The cost of offering treatment to all people currently receiving ART is already estimated to be 3% of Uganda’s Gross Domestic Product (GDP) [5, 6]. Across sub-Saharan Africa, up to 20% of total spending on health is used for HIV services, and of this over 85% is estimated to come from multilateral donors [7]. New HIV infections and need for increasing anti-retroviral treatment (ART) for HIV coverage means there will need to be a substantial increase in the projected costs of comprehensive HIV care and treatment services. Currently most HIV services are free at the point of delivery. Most people access HIV care through government health units, with some private not for profit and for profit centres offering care and treatment. However, the lack of regulation of quality of HIV services in private health care facilities means that these services are often of variable or poor quality [8, 9].

Whilst strain on health services is an ongoing concern, Uganda has a growing economy and the number of Ugandans in the middle class (defined as those whose ability to meet their basic needs is relatively stable and who can afford to save for the future) reached 12.6 million in 2012 (37% of the population) [10]. Strikingly, 89.2% of those residing in Kampala, the target area of the clinic, determined to be middle class [10] and HIV sero-prevalence increases with rising socio-economic status [2]. Those employed have a higher rate of HIV (8.0% compared to 5.0%) and those in urban compared to rural (8.7% vs. 7.0%) also have a higher prevalence of HIV. Women in the highest quintile of wealth have the highest HIV rates (5th quintile = 9.9%, 1st quintile = 6.8%) and men in the highest 2 quintiles have the highest rates of HIV (4th quintile = 8.4%, 5th quintile = 8.2%, 1st quintile = 6.3%). PLHIV in higher socio-economic groups may have easy access to private health care, however many still access this care through the public facilities. Whilst public facilities have well developed HIV services, these are often overwhelmed with long waiting times [3]. Research among women in Kampala revealed that the non-monetary costs of attending an HIV clinic visits such as a long waiting time to see a health worker led some people to choose fee for service options at nearby general clinics for conditions they otherwise would have sought care for free at their primary HIV treatment centre [11]. Studies in the US, as well as in Nigeria and Uganda suggest that delays at clinics cause customer dissatisfaction and convey a sense of poor service [12–14]. Payment for health services by the patient may counterbalance this, by allowing the patient to secure a specified set of healthcare services, at a specified level of quality and subject to a specified maximum level of personal inconvenience [15].

There have been many observational studies peformed assessing the effect of addition or removal of user fees for generalised health care systems in Africa, especially after the World Bank recommendations in the 1980s, and the majority of these show a decrease in service utilisation if fees are imposed, or an increased uptake if fees are removed [16, 17]. In HIV charging a fee for service can be associated with poor adherence to medication [18]. This data comes from situations where there are whole health services fees, which are compulsory and not discriminatory. However, we have been unable to find examples in the literature of voluntary fees being introduced for HIV services in sub-Saharan Africa as an initiative to provide an enhanced level of convenience for those who require this service.

The Infectious Diseases Institute (IDI), is a large, multi-disciplinary HIV clinic based at Mulago National Referral Hospital. Since inception, IDI has been providing patient care and treatment services free of charge but services are only available during week days between 8 am and 5 pm. The clinic caters for about 8000 PLHIV and provides general as well as specialist services such as HIV/tuberculosis (TB) clinic, young adult HIV clinic, sexual and reproductive health, most at risk population clinic (MARPS; defined in the clinic anyone who self-identifies as high risk for HIV due to occupation) and an elderly HIV clinic. In 2013 IDI started to explore alternative funding sources for the longer term sustainability of HIV services including options such as voluntary financing of some services by the PLHIV themselves in return for more convenient services. A routine client satisfaction survey carried out at IDI in May 2013, revealed that out of 387 PLHIV interviewed, 67% would like to access evening or weekend appointments, of whom 73% said they were willing to pay a fee for this extra convenience. In November 2013 a pilot After Hours Clinic (AHC) was established on Wednesday evenings requiring a co-pay fee ($16 USD; rate 1USD = 2500USh) from PLHIV to cover costs of the consultation only. Tests and drugs are provided free of charge. The purpose of this study was to ascertain opinions and perceptions about the establishment of novel inititative of a voluntary co-pay AHC within a free clinical service such as IDI.

## Methods

### Study design and sample

A cross-sectional study design using qualitative methods for data collection at IDI between 15 May 2013 to 15 June 2014 was employed. As per standard practice for qualitative methods [19] a purposeful sample of adults (over 18 years) which the team believed would answer the reserach questions was taken, including PLHIV attending the IDI clinic and IDI clinic staff. This was stratified by different participant groups including high risk, general clinic after hours, by gender and age. Focus Group Discussions (FGD) and Key Informant Interviews (KII) were conducted for the study. KII were used for PLHIV with issues of stigma, or very busy individuals who were not able to join FGD due to convience (including high net worth individuals, or health care workers). Otherwise PLHIV were invited to participate in FGDs. For FGD participation a list of ID numbers for PLHIV scheduled for a clinic visit on the day of the interview was generated and every third patient identified was approached to take part. Selection continued until 8–12 participants agreed to join each FGD. Selection of KII participants was based on accessibility and willingness to participate in the interview after a phone dicussion. IDI staff were deliberately chosen to reflect a wide range of experience and duties within the clinic. Participants were asked preference for Luganda or English for KIIs and all of the FGDs were conducted in Luganda. Data was collected by the one clinic counsellor trained in social science for KII and the same two clinic staff (one counsellor, one clinical psychologist) trained in data qualitative methods together for FGDs. These staff members had received Good Clinical Practice (GCP) training as well as training in conducting FGDs and administering individual KI interviews. They used the same study guide for each FGD and KII. The English transcripts were reviewed by a third person. The interview venues were in a private room either at IDI or at an agreed private venue convenient for the respondent in the community if KII respondents preferred.

A guide for the FGDs and IDIs was used to facilitate the discussions. These guide were piloted with two KII and two FGDs and revised prior to full data collection. The guides are available in the Additional file 1. Each FGD had a moderator and a note taker. A key informant interview guide was used in conducting KI interviews which covered similar themes to the FGDs. FGDs and KIIs were conducted in Luganda, or English. The tape-recorded interviews from FGDs and KIIs were transcribed in the language in which they were conducted and if necessary translated into English by an independent translator. FGDs lasted approximately 1 h and KII approximately 45 min.

### Data analysis

The analysis for this paper focused on attitudes and perceptions towards accessing the co-pay AHC. An explicit code book describing each category and theme was developed. Nvivo software version 10 was used to manage the data and to code each transcript according to the major domains of interest (e.g. access to care, perceptions of co-pay clinic, benefits, risks, payment). Thematic/content analysis was used. In the next step we sorted quotes based on their thematic similarities. We then examined the degree to which these themes were distributed across gender, age group and social target group. Quotations and key phrases were identified and highlighted in the findings.

### Ethical statement

The study protocol was reviewed and approved by the Scientific Review Committee (SRC) at IDI, and Makerere University School of Public Health Insitutional Review Board, as well as the Uganda National Council of Science and Technology. The study had written informed consent from participants who were informed that participation was voluntarily. Explanation of the study purpose, procedures, how long the interviews and the FGDs would last, risks and discomfort, benefits of that may arise from the study, efforts to ensure confidentiality, and voluntary nature of the study was given. Those who agreed to take part in the study were asked to sign their names or make thumbprints on the consent form.

## Results

A total of 188 participants took part in the study including IDI clinic attendees (178) and IDI staff (10). The participants were stratefied by different participant groups (e.g. accessed or not accessing after hours clinic), by gender and age (Table 1). Focus Group Discussions (FGD) with 133 participants and Key Informant Interviews (KII) with 55 individuals (10 IDI staff and 45 clinic attendees) were conducted for the study. Fourteen FGDs were undertaken; four FGDs were obtained from the general HIV clinic (30–45 years) and 10 from the IDI special clinics including: two groups of elderly (50–65 years), two of young people (18-24 years), two HIV discordant couples (30–55 years), and the sexual reproductive health (SRH) clinics (28–38 years). The final two FGDs were from Most at Risk Population (MARPS; defined in the clinic anyone who self-identifies as high risk for HIV due to occupation). We had one FGD of female commercial sex workers and one FGD for male truck drivers together with uniformed services). KII were conducted with selected 45 individuals in positions of authority and wealthy or middle class individuals. In addition, 10 IDI clinic staff members, incuding doctors, laboratory technologists, counselors, nurses, pharmacy and technicians, were also asked to participate in the KII interviews.

**Table 1:** Gender and age of study participants

Characteristic	Frequency	Percentage
Male	92	49%
Female	96	51%
Age		
18–24	18	10%
25–29	19	10%
30–34	21	11%
35–39	26	14%
40–44	34	18%
45–49	36	19%
50+	34	18%

### Attitudes and perceptions towards accessing the co-pay clinic

There were five key themes that emerged from the data regarding participant perceptions of the co-pay: 1) Benefits and reasons for accessing care at the IDI co-pay clinic, 2) Disadvantages or risks of the co-pay clinic, 3) Recommending the AHC to others, 4) Thoughts on payment for service and provision of a “Robin Hood” service, 5) Suggestions to improve service delivery.

1. Benefits and reasons for accessing care at the IDI co-pay clinic

Once at IDI, PLHIV have the choice of accessing the AHC if they wish greater convenience, quality care or perceived privacy.

“*I was referred for further care and management at IDI from Katabi H.C 111, I preferred AHC because I needed privacy and good treatment”* (KII - 46 year old female, accessing AHC).

Many PLHIV were referred to the general clinic at IDI for complex or advanced disease management.

Convenience of the AHC was often cited by many of the different groups as the reason for using the clinic. *“I like the idea of an after-hours clinic. It is what I have been yearning for*.... *I like it so much for convenience purposes”* (KII - 54 year old female, not yet accessed AHC).

Respondents thought that those in regular employment would be the main beneficiaries of the AHC since they find difficulty attending within normal clinic hours. Scheduling outside of working hours as well as reduction in waiting times are two advantages.

*“If I tell the doctors that I am from “Kasawo” (fee for service, or AHC), I don’t expect to be delayed… after triage, I know where I am going, to “Kasawo” (private)…. I just go right away. Private clinic (AHC) will reduce on waiting time in the general clinic where you just sit”* (FGD of senior citizens -65 year old female, not accessed AHC).

The AHC was also thought to benefit those who require privacy for fear of stigma.

*“Naturally people get stigmatized, evening clinic (AHC) has fought stigma a lot because you are attended to individually, and there are few people which reduced my stigma.”* (KII - 47 year old male, accessed AHC).

Participants and staff highlighted that provision of specialist services or increased time for consultation in the co-pay clinic would provide additional benefits.

“*Since it is a once a week clinic moreover on a light day, I am okay working with it. I get overtime allowance. Working in the after-hours clinic has given me a chance to interact with patients more on a one-on-one basis. It is more involving because patients are few, I create rapport with my patients and they ask questions about health”* (KII- staff– 38 year old female, physician working in AHC).

*“The beauty of this AHC is that there are specialists who will examine other illnesses other than HIV so that he or she receives sufficient treatment to improve life.”* (FGD – SRH clinic- 28 year old female, not accessed AHC).

2) Disadvantages or risks of AHC

Some participants did not agree with the fee for health service; there was fear expressed that either IDI management or wider policy makers could try to involuntarily extend fee for service to a wider group of PLHIV.

*“When there are many people who can afford to pay 20,000* ($8 USD) *you will find that management may decide to shift the general clinic to private. They will end up transferring other clients to other centers because IDI has become a private clinic in future.”* (FGD- young adults - 18 year old female, not accessed AHC).

Some PLHIV also expressed uneasiness about money being mentioned in relation to clinical services.

*“Whenever you engage money in something, it creates suspicion … We have never complained of missing drugs in the general clinic because we lack money. ...So anyone who talks about money I feel angered”* (FGD- discordant couples - 43 year old male, accessed AHC).

Fear of unequal distribution of services in the general clinic was another issue raised. They felt PLHIV in the general clinic may receive poor quality care compared to those who paid for services.

“*Those who come from” Kasawo are the ones who will see a “Musawo” (Those who have money to pay for services are the ones who can see a doctor)…. If you do not come from “Kasawo” (AHC), it means… do not grumble that your file is lost, or complain that you have spent a whole day when delayed…“the healthworkers are targeting evening clinic patients” (AHC)* (FGD- discordant couples - 50 years old female, not accessed AHC).

Ensuring equity of services despite payment was considered important.

*“There should not be discrimination in offering specialized HIV services, everyone should benefit, and givers will be motivated to give more because they are seeing an equitable distribution of their resources.”* (KII- 37 year old male, accessed AHC).

There was also concern expressed that a paid service will have a negative effect on those who are unable to afford;

*“What I do not want is the private room to be set up near the general clinic. It shows a bad picture to others. Patients will say in this private room you pay and in that you do not.… I will be emotionally affected when I come and I do not have money”.* (FGD- general clinic - 45 year male, not accessed AHC).

3) Recommending a friend to an AHC at IDI

Many participants were willing to recommend a friend or a relative, and some irrespective of HIV status:

*“I would recommend, we refer members, for example I have my mum too. She is a known cancer patient, with diabetes. We have to take her to other private clinics for quick services, given a chance I can bring her here instead of taking her to other clinics”.* (FGD- SRH- 26 year old female, not accessed AHC).

4) Thoughts on payment for service and provision of a “Robin Hood” service

A theme that came out clearly was the payment for health care services in an after hours clinic. Results revealed that PLHIV were willing to pay for service through different modes such as cash deposits, insurance or using a mobile money transfer system. The consultation fee suggested by PLHIV ranged between 5000/−USh (Uganda Shillings) (USD$2) upto 40,000/−USh shillings (approximately USD$16). The suggestions were similar usual consultation fees in different fee for service health facilities in Uganda. Some AHC users suggested that the charges could be increased.

*“40,000/- USh is 0kay for me per visit, however, should charge according to caliber of client”*. (KII- 53year old female, accessed after hours clinic).

*“No problem paying a fee for a service, in the past I suffered from a heart problem that cost me over 5 million USh* (USD$2000)*. I would pay amount of money that will restore my health.*” (KII- 59year old female, not accessed after hours clinic).

Some respondents were willing to pay for specialist consultations, branded drugs, lab tests and other services which have not been provided in the general clinic.

*“If all health services were in one place I would be willing to pay for all of them. For instance dental, optical, cancer screening, prostate cancer screening general medical examination, branded drugs*.” (KII - 55 year old male, accessed AHC).

Some respondents felt money collected could be used for additional funding for IDI of which some would be used for extention of other services in the general clinic.

“*This clinic will contribute to the income of the organization (IDI).”* (FGD- young adults - 20 year old female, not accessed AHC).

*“It justifies the cause for this payment. They pay money to pay staff. What other thing extra are the patients benefiting from? It comes in like a project. Which we are part of, our staff is part of, and it is actually for the mutual benefit of all of us who attend this clinic.”* (FGD - Senior citizens clinic – 55 year old male, not accessed AHC).

Additional funds generated could be used to complement general clinic patients health care, ideas such as food and drug purchase.

*“There are those patients who may come here for drugs but lack food. The drugs given cannot be effective without food. If they can allocate a portion of money to buy food, like porridge, that should be given to boost malnourished patients*.” (FGD- discordant couples - 39 year old female, not accessed AHC).

5) Suggestions to improve delivery

Some people expressed preference for health workers of their age range or of a particular gender;

*“Health workers should be our agemates. A mature doctor may not understand what you go through because they are past that stage…Telling a mature person that you got a girlfriend, they may not understand you.”* (FGD- young adult -21 year old male not accessed AHC).

*“I suggest both male and female should work in that clinic because one may feel more comfortable to be treated by a fellow woman or a fellow man… I can reveal my private parts to a fellow man*.” (FGD- discordant couple - 40 year old male, not accessed AHC).

Whilst the evening service was deemed convenient for many interviewed, others requested weekend services or at alternative times as well.

*“Some patients come from far distances; they may not be in position to wait until evening. Some have to pick children from school... If it is 5:00pm, it requires when you are nearby Kampala. People from Masaka cannot wait up to evening”…* (FGD- discordant couples- a 33 year old female, not accessed AHC).

Respondents felt that publicity about the AHC would be very important for future sustainability.

“*Make a brochure to circulate in other institutions. Give health radio talk-shows.*” (KII –Staff- 39 year old female, lab technician, not worked in AHC).

Poor time and human resource management was raised as an ongoing concern and a further risk anticipated when patient numbers increase.

*“I think the after hours clinic is understaffed. If the doctor is handling the file another should be on line to handle another patient. Sometimes I feel the doctors are over-worked because I presume they are the same doctors who work during day time”* (KII -37 year old male, accessed AHC).

Participants expressed frustration that the IDI clinic pharmacy had limited supplies of prescribed medication or differing brands and expressed support for the setting up of a private pharmacy at IDI which will help access drugs that are not provided for free within IDI’s current funding structure.

Flexibility in payments such as advanced payment was suggested and some expressed ideas for using new technology such as “mobile money” (money transfer through mobile phones) or using insurance schemes. Other ideas raised included paying in advance or staggering payment were suggested;

*“I think such small money requires “pay as you go”… Some modification can also be made, I can make my appointment earlier, if you put in place, mobile money to send money and I use it for booking as well”* (FGD- senior citizens clinic – 55 year male, not accessed AHC).

Flexibility with patient’s payments was encouraged and movement between co-pay and free services should be allowed;

“*If someone says they do not have the required consultation fee, do not insist, tell them that give us what you have .….. Do not emphasize on the money but the service. You can even say it is our policy, we are going to serve you but next time when you are unable to pay you are free to return to the general clinic that is free.”* (KII - 37year old male, accessed AHC).

### Themes by different groups interviewed

#### Across age

There was no difference between age groups in the perception of co-pay service; participants from all groups considered it beneficial and would recommend the service to friends, were willing to pay for service, and suggested ways to improve service delivery. Older age groups (40 years and above) who accessed co- pay services liked the privacy and agreed to pay higher amounts compared with younger people who suggested a lower fee of 5000 shillings per visit. The young adults also suggested that the age of health workers be considered and would like to be able to access a clinic at the weekends.

“*One will determine that a doctor who is going to attend to…patients in 20s is in the age bracket of 20s or 30s. The one who is going to attend to an 80 year old should be knowledgeable about the needs and demands of an 80 year old.”* (FGD- , young adults- 20 year old male, not accessed AHC).

#### Across gender

Men were very appreciative of the opening times compared to women (being after working hours). Some women had conflicting evening responsibilities e.g. picking children from school. Some respondents preferred health workers of the same sex to treat them, but others did not mind being treated by any qualified doctors.

“*I also prefer male doctors. They are more caring. They are approachable, but women oh! Laughter. Personally even when I go to the market I do not buy from women”.* (FGD, discordant couples- 38 year old female, not accessed AHC).

### Socio-economic status

The attitude towards amount to be paid was dependent on the income of the participant; some people were happy to pay any amount to receive quality services, and some were more cost conscious.

*“200,000/- USh* (USD$ 80) *is not a problem if I can offer it to other people what about my life. No problem for paying a fee for a service. I think 40,000/- per visit is low it can only pay staff. I f the resources are available helping is good idea, however, encourage friends to work”.* (KII- 51year old female, accessed after hours clinic).

Those in public office or who were health care workers were much more concerned about the stigma of being seen in clinic.

*“*I *do not like to be found in a public place like this one, I am sure it is true with other politicians, it is a challenge because they know why you are here*.” (KII - 45 year old female, accessed AHC).

### Differences in response between staff and PLHIV

PLHIV and staff were generally happy with the new set up of the clinic. Whilst health workers liked incentives such as overtime payments, transport allowance, the fewer patients per clinic. Some PLHIV however, were not keen on overtime as they felt that if the same staff members who work overtime may suffer from fatigue. Staff members were concerned about the risk of PLHIV not keeping the fixed appointments for the co-pay and suggested PLHIV be called prior to their clinic appointment. Both PLHIV and staff reported that money collected would be additional funding for IDI and that those funds could be used in the general free clinic.

*“Patients are able to get a service at their convenience, generate funds for the institution as a source of income, career building and clinical exposure, increased privacy, short waiting time. Since patients are fewer, there is attention to detail, chances of choosing specific doctors, staff-patient relationship, staff given per diem”.* (KII- staff – 39 year old female, lab technologist not worked in after hour’s clinic).

## Discussion

This study used the opportunity of the development of a new service to undertake a qualitative evaluation of PLHIV and staff perspectives on this service. The authors feel this is an important contribution to the literature as we have been unable to find any other published work related to patient experiences of an out of hours service or co-pay services nested within free services for HIV in sub-Saharan Africa. Furthermore, there were very few qualitative articles exploring perceptions of user fees more generally. Therefore, we feel that this paper is an important contribution to the discussion about different models of sustaining HIV services in Uganda and beyond as the number of people we need to treat continues to rise.

This study was undertaken during the early phases of the establishment of a convenient clinic with a co-pay charge for HIV services. The clinic was designed to provide the same care to PLHIV as in the general clinic; the same clinic staff, the same drugs and the same tests. The only planned difference was the provision of services out of hours in order for PLHIV to access care at a time convenient to them.

In general there was a positive response by PLHIV and staff to the AHC and acceptability of payment for services provided at convenient times. The PLHIV and clinicians observed that the AHC model also allows PLHIV to have more time with their clinicians and are able to explore non-HIV related conditions. Additional benefits highlighted in the interviews included an avoidance of stigma through increased privacy afforded by a quieter clinic, and that income generated could help to provide services for PLHIV with more limited financial resources. Work on barriers to HIV services in South Africa highlighted inconvenient clinic hours, long clinic queues, not being able to get appointments, and disrespect as barriers to care, as well as stigma as issues affecting access to care [20]. In Zimbabwe patients also expressed that longer waiting times were a cause of stress [21]. In the South African patients preferred to attend semi-private clinics which charged for some services (as compared to free public clinics), as wait times were shorter, and quality of care was perceived to be higher This is in line with our findings at the AHC clinic, although our model differs by combining AHC services in a free clinic.

Payment for services (user fees) for health care in Africa are controversial. They were introduced to many sub-Saharan African countries after World Bank guidance in the 1980s and 90s [16], but were subsequently dropped from many countries due to concerns of decreased utilization of services after introduction [22–26]. Following the discontinuation of fees there was some increase in health care utilization, but these effects are difficult to quantify due to challenges in studying these large scale health system changes [17]. In line with these findings for those with HIV in Uganda, data shows that when PLHIV were paying for ART drugs, having a low monthly income was a risk of low adherence [18]. A study of ART adherence undertaken in Kampala as free ART was being rolled out, showed a 3.8% increase in adherence in those who received free treatment [27]. Not all user fee introduction was associated with decreased utilization. There are two studies of the introduction of user fees suggested that when user fees were introduced as well as quality improvements to the service, the utlisation of services did not decrease [28, 29]. The respondents (staff and patients) in this study stated that there was an (unintended) improvement in services with the introduction of the AHC; mainly in relation to better clinic time management with longer appointment times and less waiting time, as well as managing non HIV related conditions. During the user fee roll out in Uganda, data from rural settings showed that clinic attendees welcomed payment of staff incentives [30]. Our study shows that staff welcomed the overtime incentive to increase their basic pay, but PLHIV discussed the benefit to the whole institution rather than individual staff.

In our study, the major concern raised by the PLHIV about the clinic is related to a sense of opening the door for privatization of care more generally at IDI and beyond. This is understandable given the recent history of user fees and payment for ART in Uganda. Whilst the AHC model is very different to the previous payment for services (out of hours only, voluntary payment, free drugs and tests), it was still seen as a possible ‘slippery slope’ to full re-introduction of user fees. The authors do not support full scale re-introduction of users fees and are keen to emphasize that this clinic was an arrangement designed to help to sustain services for those PLHIV less able to pay, and not as a replacement of free care to those who require it. The AHC is nested within a free clinic, and PLHIV can easily move from the co-pay service to the free service when need arises, which was seen as another positive benefit by study respondents.

A report of the effect of implementation of user fees for health in Uganda showed that whilst there were negative effects on the low income health care users, there were no negative effects on higher income users [31]. The IDI AHC clinic is targeting higher income users, and study respondents in this group found the charges acceptable, and in some cases suggested an increase. The demographics of the HIV epidemic in Uganda show that the urban highest socio-economic classes have the highest HIV prevalence [2]. It is therefore imperative that we provide access to HIV services for this group which fit around their lifestyle. Our AHC preliminary data suggests that there has in fact been a positive effect on patient outcomes in this clinic, especially those who were poorly adherent before joining the co-pay clinic [32]. Whilst we were unable to find published qualitative work in a similar clinical setting to the AKC, as study from Ethiopia, across health services where some patients paid and some didn’t showed that unemployed persons are more likely to rate their HIV as high quality than those with a job. This may be related to raised expectations, as well as lack of convenience for those who are working, similar to what we have found in our qualitatative work [33].

However, another concern expressed is an addition of a two tier system with some PLHIV who can afford to pay getting better services. This is a concern as the AHC PLHIV are expressing that the service is improved especially in regards to time management. De-congesting the general clinic by offering out of hours appointments through the AHC should improve care for both clinics. Since the study, we have added a cheaper AHC clinic run by less senior doctors that runs on a different day and is costed at US$5 per visit. This is an attempt to provide a range of co-pay and free services so that people with different budgets can access out of hours care. A co-pay pharmacy has also been opened which offers an extended formulary to all IDI PLHIV at a cheaper price than standard private pharmacy prices, as informed by respondent needs;

*“The doctor may prescribe drugs, when you reach the pharmacy window, they tell you that some drugs are not available, go buy them in a private pharmacy. Even some lab services, there are some which are not readily available here, when it necessitates you to go to some other bigger private laboratories”.* (FGD- senior citizens- clinic –45year old male, not accessed AHC).

There was a general acceptance amongst those who were paying for services that the payment should be used to directly sustain the clinic for PLHIV with fewer financial resources. There is some data to show that the cost of management of a co-pay service alongside a free service can cancel out any financial gains [34], and so we will be undertaking a cost benefit analysis of the AHC clinic to understand if this is happening in the AHC. As suggested by the study respondents if there is excess revenue generated we will explore best use of these resources including opportunities for resource generating projects for poorer clinic PLHIV.

One limitation of the study is that the clinic had not been running for very long and so there small number of PLHIV that had accessed the after hours clinic and this group may have some bias as the first users (e.g. a relation of clinic staff, or be very well known to clinic staff). In particular KII participants in particular were selected on the basis that they had the educational ability and interest to give full responses to our questions. As the clinic was in the start up phase at the time of the study; it would be interesting to repeat the study now that the clinic is well established. The study took place in a well regarded HIV clinic based at the Mulago National Referral hospital. It is unclear if PLHIV would be willing to pay for services at other regional, rural or government centres. Social desirability bias within this cohort was possible, as the interviewers were also counselors (health care workers) in the clinic, however, the received responses were open and honest in terms of giving negative as well as positive feedback which leads us to belive that social desirability was minimal.

## Conclusion

Sustaining HIV services long term is an urgent issue for sub-Saharan Africa, as we need to provide life long treatment for over 8 million people across the continent. We believe that this is the first qualitative study of the introduction of a co-pay service nested within a free service, targeting PLHIV who have financial resources but limited time available. The experience of these PLHIV and staff caring for them is positive, with respect to convenience, decreased stigma, freeing of resources for less financially stable PLHIV. Concerns are mainly around ensuring this does not lead to a re-introduction of user fees for all. Payment for convenience of services by more financially well off PLHIV is one possible tool that can be employed to increase ART coverage; but safeguarding the resources generated to ensure that they are used to sustain care for others who are less fortunate is essential. We would encourage other providers to explore hybrid models such as our AHC, that allow for clinic space and human resources to be utilized efficiently and to provide convenient services for all PLHIV.

## Additional file

Additional file 1: Focus Group Discussion and Key Informant Interview Guides. Focus Group Discussion and Key Informant Interview Guides. (RTF 83 kb)

## Abbreviations

AHC: After hours clinic
AIDC: Adult Infectious Diseases Clinic
AIDS: Acquire
ART: Antiretroviral Therapy
d: ImmuneDeficiency Syndrome
FGD: Focus Group Discussions
GCC: Grand Challenges Canada
GCP: Good Clinical Practice
GDP: Gross Domestic Product
HIV: Human Immunodeficiency virus
IDI: Infectious Diseases Institute
IRB: Institutional Review Board
KII: Key informant interviews
MARPS: Most At Risk Populations
MOH: Ministry of Health
PLHIV: People living with HIV
SRC: Scientific Review Committee
SRH: Sexual Reproductive Health
TB: Tuberculosis
UAC: Uganda AIDS Commission
USD: United States Dollar USD
WHO: World Health Organisation.
