# Supplementary material for: Exploring attitudes and perceptions of patients and staff towards an after-hours co-pay clinic supplementing free HIV services in Kampala, Uganda
Source: BMC Health Serv Res. 2017 Aug 22;17:580. doi: 10.1186/s12913-017-2524-5 (PMC5568083; doi:10.1186/s12913-017-2524-5)
Supplement: Additional file 1: — Focus Group Discussion and Key Informant Interview Guides. Focus Group Discussion and Key Informant Interview Guides. (RTF 83 kb) [file 12913_2017_2524_MOESM1_ESM.rtf]

Focus Group Discussion and Key Informant Interview Guides

After Hours (AHC)
Elderly/Discordant couples/ young people/Most At Risk population/General HIV clinic patients
 Focus group discussion.

Introduction 
Greetings……..You are welcome to this focus group discussion. My name is…….. We are asking you to participate in the study related to “attitudes and perceptions towards establishing an after hours or weekend clinic at IDI. We are aware that some patients may be willing to pay a fee for care to be received at more convinient time for them.We expect to get your ideas during this  focus group discussion concerning this matter and this will enable IDI administrators in proper planning of that clinic. The findings from this study will be confidential and nothing you say will offend us. We need your ideas for this to be a success. There will be a discussion moderator and  a note taker, the discussion will last one hour. The study staff will both take notes and record your voices, this will allow us to fill in the gaps we may have missed during note taking. You are requested to speak audibly enough so all can hear and to take turns in speaking to enable us note down your ideas. How many of you will allow us to voice recoder ? As we are tape recording the interview, we ask that you refrain from using names or identifying information of yourself or your partners. If at any time during the interview you feel uncomfortable you can ask for a break, refuse to answer any question, and are always free to leave. Do you have any questions before we start? 

Section A-Demographics.
Date  of interview …………….Place of discussion ………………………………
Moderator …………………………….. Secretary ………………………
Language used  to conduct the interview…………………………………..
Start time ……………………….. End time…………………….
Gender of interviwees……………………………………….
Interviewee Numbers…………………………………………………..
Age range…………….. 


Section B.  General experience  of FGD members at IDI.
1.	As an elderly/ Discordant couple /young person/xx / Breast feeding mothers (fill in appropriate), what led you to receive care at IDI and not any where else?
2.	What challenges do you find while receiving care at IDI? (Probe for personal, staff, and facility).
How  easy is it for you to keep clinic appointments? (probe for clinic timing)
3.	How do you feel when your acquantices see you come to the clinic? (probe for stigma)
Section C. Perceptions  and attitudes regarding an after hours or weekend clinic.
5.	What are your thoughts regarding a clinic that would take place after hours or weekends at IDI? (probe for willingness to pay for convenient services).
6.	What are your thoughts regarding scheduling of appointments for evening, weekend or any other scheduled times for you to return and be seen ?
7.	What advantages or disadvantages may a person  face receive when they attend AHC or weekend clinic? (probe for likes and dislikes).
8.	What time would you recommend to open and close an evening clinic and week end clinic ?
Section D.  Healthcare Services in an after hours  or weekendclinic.
9.	If you agree to be seen during after hours clinic would you agree to pay a fee for this ? (probe for willingness to pay)
10.	If we offer care to you during after hours clinic what illnesses should we treat other than HIV ? (Probe for Hypertension, Diabetics, and Arthritis)
11.	What type  of health workers  should work in the after hours or weekend clinic? (Probe for category, qualification, experience, age).
12.	What are your thoughts regarding paying a fee for your health services (Probe for what they would want to pay for eg. Blood tests, doctor's consultation,, counselor etc.).
13.	Personally, what would  you want to pay for ?
14.	What  do you propose to be the mode of payment for an evening/ weekend clinic ?) (Probe ; Monthly fee, on each appointment, for each service ?)
15.	In your opinion which additional  services would you be willing to pay from the AHC which may not be provided by the free clinic ?
16.	How much would you be willing to pay for those after hours or weekend services? (Probe for amount and mode of payment).
17.	Would you  recommend a friend or relative to attend an after hours  or weekend clinicat IDI?
18.	Could part of the fee you pay help care for the underpriveleged (poor) attending the general clinic ?
19.	Is there any other health related service that you donot receive at IDI currently and would be willing to pay for ? (Probe for additional  services required).
20.	Is there any other health related services that you are currently paying for outside IDI? (Probe for services paid outside).
Section. E. Suggestions towards enhancing the quality of healthcare deliverly. 
21.	 What are your suggestions on what can be done to have excellent clinic care at IDI and specifically at an after hours or weekend clinic ?

Thank you.


After Hours (AHC)
Key Informants:  IDI staff working or not working in AHC/ Special IDI patients attending and not attending AHC. 
Key Informant (KI) guide.

Introduction.
Hello, my name is _____________ and I want to thank you for joining us today. I am helping to coordinate this study on after hours clinic (AHC) here in IDI. We are conducting this research to identify your views about AHC; we are asking you to participate in the study related to “attitudes and perceptions towards accessing an after hours or weekend clinic at IDI. I would like to say that there are no right or wrong answers in our conversation. We will simply be discussing your views, opinions and experiences on a range of topics, so please feel comfortable to say how you honestly feel. I would like to tape record the whole session. Please do not be concerned about this: all measures will be taken by the researchers to maintain confidentiality of the interviews and discussions. Information you tell us will ONLY be used for this research project. As we are tape recording the interview, we ask that you refrain from using names or identifying information of yourself. If at any time during the interview you feel uncomfortable you can ask for a break, refuse to answer any question, and are always free to leave. Do you have any questions before we start? 

Section A.  Socio demographic factors 
Date …………………….. ID No……………………………….
1.	Sex of the respondent …………………………………………..
2.	Position of the respondent (Job title)……………………..
Section B.  General experience  of  Key informant at IDI.
3.	As a xx (fill in appropriate), What are reasons for patients seeking care at IDI and not any other clinic? (For those accesing AHC, probe  for what prompted them to attend care)
4.	What challenges have you faced while accessing care at IDI? (Probe for personal, health worker related and facility related, faced also by patients ? 
5.	How convenient is the timing for clinic appointments? (probe whether timing is difficult in general clinic or AHC)
6.	How do you feel being seen by people you may  know in the community at the clinic? (probe for stigma in general or AHC)
Section C. Perceptions and attitudes regarding an after hours or week end clinic at IDI.
7.	Tell me what you know about an after hours clinic at IDI. (Probe whether client attends the  AHC at IDI and how long they have been in that care)
8.	What is your opinion about accessing an after hours or weekend  clinic at IDI? (For those attending AHC, probe for Impression about clinic)
9.	What is your view about having an evening, weekend or strictly timed appointment for care at IDI? (Probe for those attending AHC or not)
10.	 What  benefits do you see in an  of an after hours or weekend clinic at IDI and what risks or disadvantages do you see ? (Probe for likes /dislikes).
11.	When would you want the after hours or week end clinic open and accessible to you ((probe for appropriate time or day).
Section D. Health care Services in an after hours clinic at IDI.
12.	 If you agree to be seen during after hours clinic would you agree to pay a fee for this ? (probe for willingness to pay)
13.	If we offer care to you during after hours clinic what illnesses should we treat other than HIV ? (Probe for Hypertension, Diabetics, and Arthritis).
14.	What type  of health workers  should work in the after hours or weekend clinic? (Probe for category, qualification, experience, age)
15.	What are your thoughts regarding paying a fee for your health services (Probe for what they would want to pay for eg. Blood tests, doctor's consultation,, counselor  etc).
16.	Personally, what would  you want to pay for ? (Probe for view about the fee charged for consultation t.
17.	What do you propose to be the mode of payment for an evening/ weekend clinic ?) (Probe ; Monthly fee, on each appointment, for each service, for those attending).
18.	In your opinion which additional services would you be willing to pay from the Convenience CoPay Clinic which may not be provided by the free clinic ?
19.	How much would you be willing to pay for those after hours or weekend services? (Probe for fixed fee per clinic visit, which services to pay for).
20.	Would you  recommend a friend or relative to attend an after hours  or weekend clinic at IDI? 
21.	Could part of the fee you pay help care for the underpriveleged (poor) attending the general clinic ?
22.	Is there any other health related service that you donot receive at IDI currently and would be willing to pay for ? (Probe for additional services to pay for).
23.	Is there any other health related services that you are currently paying for outside IDI? (Probe for services paid  for before joining pilot AHC).
Section. E. Suggestions towards enhancing the quality of healthcare deliverly.
24. What are your suggestions on what can be done to have excellent clinic care at IDI and specifically at an after hours or weekend clinic ? 

Thank You.
